# Supplementary material for: Mass bathing events in River Kshipra, Central India- influence on the water quality and the antibiotic susceptibility pattern of commensal E.coli
Source: PLoS One. 2020 Mar 4;15(3):e0229664. doi: 10.1371/journal.pone.0229664 (PMC7055887; doi:10.1371/journal.pone.0229664)
Supplement: S1 Data — (DOCX) [file pone.0229664.s001.docx]

**Supplementary Data**

Table S1. Description of sampling sites.

|  |
| --- |
| **Sampling Point No 1: Kshipra (Triveni),** 23^0^ 07’42.516” N latitude and 75^0^ 47’ 47.513” E longitude**):** This is located on the River Kshipra where River Kshipra enters the Ujjain city. This site is situated at upstream of confluence of River Kshipra and River Khan. The maximum depth at this site is about 2 to 3 meter with weed infested banks. Flood plain area of right and left banks is dominated by agricultural activities. |
|  |
| **Sampling Point No 2: Ramghat (**23^0^ 11’21.930” N latitude and 75^0^ 45’ 54.180” E longitude)**:** This is located at thecentre of the Ujjain city. This site is the most important religious site and receives maximum number of devotees. Both banks are used for bathing, washing and boating activities all the year round and more often during mass-bathing occasions. The maximum depth at this site is about 2 to 3 meter. |
|  |
| **Sampling Point No 3: Kaliyadeh (**23^0^ 14’39.706” N latitude and 75^0^ 46’ 55.817” E longitude)**:** This is the last point before river exits from Ujjain city. Flood plain area of right and left banks is dominated by agricultural activities. The maximum depth at this site is 0.4 to 0.8 meter. |

Table S2. Water quality standards.

| **Waterquality parameter** | **Standard** | **Source** |
| --- | --- | --- |
| pH | 5 – 9 | (1) |
| Water temperature(^o^C) | 32 | (2) |
| Conductivity(µS/cm) | 1000 | (1) |
| Total dissolved solids (mg/L) | 500 | (2) |
| Turbidity(NTU) | 10 | (2) |
| Total Alkalinity(mg/L) | 30 to 90 | (1) |
| Chloride(mg/L) | 230 | (1) |
| Total Hardness(mg/L) | 300 | (2) |
| Nitrate Nitrogen (mg/L) | 10 | (1) |
| Total Phosphorus(mg/L) | 0.01 | (1) |
| Sulphate(mg/L) | 400 | (3) |
| Dissolved oxygen(mg/L) | 5 | (3) |
| Biochemical Oxygen Demand (mg/L) | 8 | (2) |
| Chemical Oxygen Demand(mg/L) | 20 | (2) |
| Total Coliform  CFU/100 ml | 50 or less | (4) |
| Total *E. coli*  CFU/100 ml | Shall not be detectable | (4) |

Table S3. Sediment quality of river Kshipra during mass gathering.

| **Sampling Point Name** | **Period/Time** | **Code** | **DOS** | **pH** | **Soluble Bicorbonate (mg/kg)** | **Chloride (mg/kg)** | **Nitrate- Nitrogen (mg/g.)** | **Available Phosphorus (mg/g.)** | **Organic Matter (%OM)** | **TCC/100ml** | **TEC/100ml** |
| --- | --- | --- | --- | --- | --- | --- | --- | --- | --- | --- | --- |
| Triveni Ghat | Pre | ORS011408007 | 24/08/2014 | 8.0 | 716.29 | 119.88 | 2.402 | 1.0468 | 0.797 | 550 | 60 |
| Triveni Ghat | During | ORS011408008 | 25/08/2014 | 7.8 | 695.76 | 109.89 | 2.410 | 1.0936 | 0.687 | 18000 | 500 |
| Triveni Ghat | Post | ORS011408009 | 25/08/2014 | 7.7 | 648.76 | 114.885 | 2.392 | 1.0752 | 0.687 | 28250 | 2750 |
| Ramghat | Pre | ORS 011408001 | 24/08/2014 | 7.5 | 773.05 | 99.9 | 3.293 | 1.3648 | 1.456 | 42800 | 5200 |
| Ramghat | During | ORS 011408002 | 25/08/2014 | 7.4 | 721.59 | 159.84 | 3.378 | 1.4692 | 1.703 | 56000 | 10900 |
| Ramghat | Post | ORS011408003 | 25/08/2014 | 7.6 | 757.34 | 109.89 | 3.418 | 1.5146 | 0.494 | 28000 | 1500 |
| Kaliyadeh | Pre | ORS 011408004 | 24/08/2014 | 7.5 | 645.01 | 114.885 | 1.404 | 0.2172 | 0.797 | 12000 | 100 |
| Kaliyadeh | During | ORS 011408005 | 25/08/2014 | 7.5 | 712.88 | 109.89 | 1.273 | 0.2048 | 1.263 | 16000 | 0 |
| Kaliyadeh | Post | ORS 011408006 | 25/08/2014 | 7.3 | 647.55 | 154.845 | 1.274 | 0.2072 | 1.456 | 9750 | 2500 |
| Triveni Ghat | Pre | ORS021411016 | 21/11/2014 | 6.6 | 587.02 | 179.82 | 2.906 | 1.2492 | 0.687 | 110000 | 1000 |
| Triveni Ghat | During | ORS021411017 | 22/11/2014 | 6.1 | 486.25 | 114.885 | 2.807 | 1.4452 | 0.797 | 216000 | 6000 |
| Triveni Ghat | Post | ORS021411018 | 22/11/2014 | 5.6 | 386.60 | 94.905 | 3.022 | 1.6528 | 0.824 | 506000 | 6200 |
| Ramghat | Pre | ORS 021411010 | 21/11/2014 | 6.7 | 1323.47 | 119.88 | 2.346 | 1.0936 | 1.126 | 3370000 | 7000 |
| Ramghat | During | ORS 021411011 | 22/11/2014 | 7.2 | 1199.40 | 104.895 | 2.360 | 1.1772 | 0.742 | 140000 | 800 |
| Ramghat | Post | ORS021411012 | 22/11/2014 | 7.3 | 982.26 | 99.9 | 2.433 | 1.0692 | 0.549 | 4440000 | 6000 |
| Kaliyadeh | Pre | ORS 021411013 | 21/11/2014 | 7.0 | 992.67 | 109.89 | 1.529 | 0.247 | 1.346 | 126000 | 100 |
| Kaliyadeh | During | ORS 021411014 | 22/11/2014 | 7.3 | 1021.93 | 1243.755 | 1.542 | 0.2296 | 0.961 | 220000 | 1000 |
| Kaliyadeh | Post | ORS 021411015 | 22/11/2014 | 7.1 | 1034.15 | 104.895 | 1.406 | 0.2832 | 1.099 | 196000 | 1000 |
| Triveni Ghat | Pre | ORS031504025 | 17/04/2015 | 7.9 | 365.63 | 95.26524 | 3.030 | 2.0936 | 0.405 | 24000000 | 280000 |
| Triveni Ghat | During | ORS031504026 | 18/04/2015 | 7.6 | 418.47 | 90.25128 | 3.032 | 2.4938 | 1.165 | 12000000 | 1300 |
| Triveni Ghat | Post | ORS031504027 | 18/04/2015 | 7.4 | 385.02 | 105.29316 | 3.211 | 2.4996 | 1.950 | 18000000 | 5200 |
| Ramghat | Pre | ORS031504019 | 17/04/2015 | 7.6 | 362.44 | 107.80014 | 3.457 | 1.7892 | 0.810 | 4300000 | 500 |
| Ramghat | During | ORS031504020 | 18/04/2015 | 7.3 | 332.01 | 102.78618 | 3.553 | 1.6912 | 1.241 | 30000000 | 1000000 |
| Ramghat | Post | ORS031504021 | 18/04/2015 | 7.5 | 303.90 | 105.29316 | 3.552 | 2.0938 | 1.114 | 84000000 | 8700 |
| Kaliyadeh | Pre | ORS031504022 | 17/04/2015 | 7.4 | 390.63 | 47.63262 | 1.570 | 0.6336 | 0.380 | 11800000 | 400 |
| Kaliyadeh | During | ORS031504023 | 18/04/2015 | 7.6 | 421.21 | 55.15356 | 1.548 | 0.5538 | 0.329 | 1,00,00,000 | 4700 |
| Kaliyadeh | Post | ORS041505024 | 18/04/2015 | 7.4 | 390.05 | 57.66054 | 1.687 | 0.5728 | 0.304 | 58000000 | 8500 |
| Triveni Ghat | Pre | ORS041505034 | 17/05/2015 | 7.6 | 778.21 | 85.23732 | 3.324 | 1.9752 | 0.613 | 210000 | 8000 |
| Triveni Ghat | During | ORS041505035 | 18/05/2015 | 7.4 | 689.16 | 105.29316 | 3.404 | 1.9732 | 0.581 | 80000 | 0 |
| Triveni Ghat | Post | ORS041505036 | 18/05/2015 | 7.7 | 671.14 | 90.25128 | 3.361 | 1.9592 | 0.710 | 240000 | 30000 |
| Ramghat | Pre | ORS041505028 | 17/05/2015 | 7.5 | 352.23 | 115.32108 | 3.064 | 2.2528 | 1.162 | 23000000 | 3000000 |
| Ramghat | During | ORS041505029 | 18/05/2015 | 7.2 | 356.48 | 130.36296 | 2.934 | 2.597 | 2.615 | 2360000 | 370000 |
| Ramghat | Post | ORS041505030 | 18/05/2015 | 7.3 | 277.26 | 127.85598 | 3.045 | 2.6032 | 2.163 | 3800000 | 210000 |
| Kaliyadeh | Pre | ORS041505031 | 17/05/2015 | 7.8 | 416.07 | 55.15356 | 1.486 | 0.3778 | 0.420 | 108000 | 1000 |
| Kaliyadeh | During | ORS041505032 | 18/05/2015 | 7.6 | 375.59 | 57.66054 | 1.658 | 0.2952 | 0.323 | 40400000 | 28400000 |
| Kaliyadeh | Post | ORS041505033 | 18/05/2015 | 7.7 | 401.51 | 55.15356 | 1.660 | 0.3646 | 0.452 | 340000 | 110000 |
| Triveni Ghat | Pre | ORS051510043 | 11/10/2015 | 8.3 | 592.27 | 115.32108 | 5.782 | 3.3524 | 1.503 | 299000 | 6000 |
| Triveni Ghat | During | ORS051510044 | 12/10/2015 | 7.8 | 561.62 | 105.29316 | 6.152 | 3.1734 | 1.227 | 88000 | 13000 |
| Triveni Ghat | Post | ORS051510045 | 12/10/2015 | 8.1 | 576.18 | 127.85598 | 6.029 | 3.0936 | 1.319 | 162000 | 68000 |
| Ramghat | Pre | ORS051510037 | 11/10/2015 | 8.6 | 587.00 | 115.32108 | 5.032 | 3.7772 | 0.859 | 1580000 | 15500 |
| Ramghat | During | ORS051510038 | 12/10/2015 | 7.9 | 463.38 | 132.86994 | 5.278 | 4.4336 | 1.197 | 143500 | 28000 |
| Ramghat | Post | ORS051510039 | 12/10/2015 | 8.2 | 532.89 | 135.37692 | 5.266 | 4.4936 | 1.135 | 146000 | 5500 |
| Kaliyadeh | Pre | ORS051510040 | 11/10/2015 | 8.3 | 274.18 | 65.18148 | 1.698 | 1.1296 | 0.614 | 14000 | 2000 |
| Kaliyadeh | During | ORS051510041 | 12/10/2015 | 8.3 | 290.14 | 60.16752 | 1.656 | 1.0332 | 0.798 | 110000 | 14000 |
| Kaliyadeh | Post | ORS051510042 | 12/10/2015 | 8.4 | 302.81 | 62.6745 | 1.667 | 1.0436 | 0.276 | 104500 | 1500 |
| Triveni Ghat | Pre | ORS061511052 | 24/11/2015 | 7.9 | 703.81 | 105.29316 | 4.333 | 2.9738 | 0.301 | 288000 | 3000 |
| Triveni Ghat | During | ORS061511053 | 25/11/2015 | 7.8 | 679.07 | 122.84202 | 4.285 | 2.9764 | 0.903 | 155000 | 16000 |
| Triveni Ghat | Post | ORS061511054 | 25/11/2015 | 7.6 | 673.19 | 120.33504 | 4.158 | 2.8714 | 0.711 | 138000 | 33000 |
| Ramghat | Pre | ORS061511046 | 24/11/2015 | 8.2 | 825.35 | 145.40484 | 4.861 | 3.2938 | 0.328 | 186000 | 46000 |
| Ramghat | During | ORS061511047 | 25/11/2015 | 7.6 | 790.62 | 155.43276 | 4.717 | 3.8512 | 0.438 | 157500 | 5500 |
| Ramghat | Post | ORS061511048 | 25/11/2015 | 7.8 | 806.02 | 140.39088 | 4.708 | 3.9536 | 0.574 | 209000 | 64000 |
| Kaliyadeh | Pre | ORS061511049 | 24/11/2015 | 8.1 | 359.34 | 65.18148 | 2.317 | 0.8452 | 0.164 | 232000 | 5000 |
| Kaliyadeh | During | ORS061511050 | 25/11/2015 | 8 | 321.89 | 60.16752 | 2.184 | 0.8498 | 0.301 | 102000 | 30500 |
| Kaliyadeh | Post | ORS061511051 | 25/11/2015 | 7.8 | 293.69 | 57.66054 | 2.308 | 0.4436 | 0.574 | 149500 | 1500 |
| Triveni Ghat | Pre | ORS071601061 | 08/01/2016 | 7.8 | 437.92 | 140.39088 | 3.570 | 1.9316 | 0.506 | 87000 | 30000 |
| Triveni Ghat | During | ORS071601062 | 09/01/2016 | 7.5 | 422.61 | 132.86994 | 3.781 | 2.0492 | 1.249 | 127500 | 47500 |
| Triveni Ghat | Post | ORS071601063 | 09/01/2016 | 7.3 | 410.06 | 135.37692 | 3.735 | 2.053 | 1.190 | 96500 | 6500 |
| Ramghat | Pre | ORS071601055 | 08/01/2016 | 8 | 631.55 | 157.93974 | 4.521 | 2.2472 | 0.625 | 96000 | 0 |
| Ramghat | During | ORS071601056 | 09/01/2016 | 7.8 | 623.80 | 160.44672 | 4.523 | 2.2852 | 0.803 | 60000 | 0 |
| Ramghat | Post | ORS071601057 | 09/01/2016 | 8.1 | 650.76 | 157.93974 | 4.471 | 2.225 | 0.654 | 129000 | 7000 |
| Kaliyadeh | Pre | ORS071601058 | 08/01/2016 | 7.8 | 231.59 | 70.19544 | 1.284 | 0.8472 | 0.357 | 90000 | 21500 |
| Kaliyadeh | During | ORS071601059 | 09/01/2016 | 7.9 | 217.81 | 65.18148 | 1.233 | 0.8448 | 0.297 | 121000 | 49000 |
| Kaliyadeh | Post | ORS071601060 | 09/01/2016 | 7.8 | 243.97 | 80.22336 | 1.557 | 0.8366 | 0.357 | 77500 | 16000 |
| Triveni Ghat | Pre | ORS081602070 | 07/02/2016 | 7.6 | 355.77 | 135.37692 | 3.783 | 2.2536 | 0.468 | 242000 | 33000 |
| Triveni Ghat | During | ORS081602071 | 08/02/2016 | 7.4 | 368.38 | 130.36296 | 3.582 | 2.0692 | 0.351 | 272000 | 50000 |
| Triveni Ghat | Post | ORS081602072 | 08/02/2016 | 7.2 | 336.70 | 135.37692 | 3.583 | 2.0456 | 0.672 | 313000 | 23000 |
| Ramghat | Pre | ORS081602064 | 07/02/2016 | 7.8 | 520.99 | 160.44672 | 4.271 | 1.8936 | 0.351 | 176000 | 44000 |
| Ramghat | During | ORS081602065 | 08/02/2016 | 7.2 | 494.74 | 155.43276 | 4.196 | 1.9772 | 0.526 | 258000 | 38000 |
| Ramghat | Post | ORS081602066 | 08/02/2016 | 7.4 | 477.15 | 155.43276 | 4.183 | 1.9848 | 0.555 | 260000 | 2000 |
| Kaliyadeh | Pre | ORS081602067 | 07/02/2016 | 8.3 | 333.52 | 80.22336 | 1.546 | 0.6528 | 0.205 | 198000 | 3000 |
| Kaliyadeh | During | ORS081602068 | 08/02/2016 | 8.3 | 356.26 | 72.70242 | 1.407 | 0.6372 | 0.263 | 276000 | 0 |
| Kaliyadeh | Post | ORS081602069 | 08/02/2016 | 8.2 | 281.80 | 75.2094 | 1.410 | 0.6912 | 0.175 | 254000 | 2000 |

Table S4. Total coliform and fecal coliform of surface water during mass gathering in river Kshipra.

| **SPN** | **Period** | **Code** | **TCC/100ml** | **TEC/100ml** |
| --- | --- | --- | --- | --- |
| Triveni Ghat | Pre | ORW011408004 | 2400 | 100 |
| Triveni Ghat | Pre | ORW011408013 | 10900 | 300 |
| Triveni Ghat | During | ORW011408005 | 28400 | 500 |
| Triveni Ghat | During | ORW011408014 | 21000 | 9000 |
| Triveni Ghat | Post | ORW011408006 | 236000 | 10000 |
| Triveni Ghat | Post | ORW011408015 | 300000 | 83000 |
| Ramghat | Pre | ORW011408001 | 68600 | 40500 |
| Ramghat | Pre | ORW011408010 | 41600 | 13600 |
| Ramghat | During | ORW011408002 | 51200 | 700 |
| Ramghat | During | ORW011408011 | 40800 | 10000 |
| Ramghat | Post | ORW011408003 | 593000 | 11000 |
| Ramghat | Post | ORW011408012 | 102000 | 8000 |
| Kaliyadeh | Pre | ORW011408007 | 32400 | 1100 |
| Kaliyadeh | Pre | ORW011408016 | 39000 | 1200 |
| Kaliyadeh | During | ORW011408008 | 32900 | 3600 |
| Kaliyadeh | During | ORW011408017 | 60800 | 20000 |
| Kaliyadeh | Post | ORW011408009 | 1520000 | 15000 |
| Kaliyadeh | Post | ORW011408018 | 304000 | 18000 |
| Triveni Ghat | Pre | ORW021411022 | 41400 | 600 |
| Triveni Ghat | Pre | ORW021411031 | 52000 | 1000 |
| Triveni Ghat | During | ORW021411023 | 380000 | 600 |
| Triveni Ghat | During | ORW021411032 | 54000 | 1000 |
| Triveni Ghat | Post | ORW021411024 | 520000 | 1300 |
| Triveni Ghat | Post | ORW021411033 | 315000 | 1000 |
| Ramghat | Pre | ORW021411019 | 28000 | 0 |
| Ramghat | Pre | ORW021411028 | 32300 | 300 |
| Ramghat | During | ORW021411020 | 3200 | 100 |
| Ramghat | During | ORW021411029 | 29000 | 100 |
| Ramghat | Post | ORW021411021 | 120000 | 100 |
| Ramghat | Post | ORW021411030 | 303000 | 1000 |
| Kaliyadeh | Pre | ORW021411025 | 21600 | 100 |
| Kaliyadeh | Pre | ORW021411034 | 35300 | 3200 |
| Kaliyadeh | During | ORW021411026 | 21200 | 900 |
| Kaliyadeh | During | ORW021411035 | 29800 | 900 |
| Kaliyadeh | Post | ORW021411027 | 35300 | 100 |
| Kaliyadeh | Post | ORW021411036 | 180000 | 2000 |
| Triveni Ghat | Pre | ORW031504040 | 107000 | 2000 |
| Triveni Ghat | Pre | ORW031504049 | 122000 | 2000 |
| Triveni Ghat | During | ORW031504041 | 141000 | 5000 |
| Triveni Ghat | During | ORW031504050 | 181000 | 4000 |
| Triveni Ghat | Post | ORW031504042 | 1100000 | 26000 |
| Triveni Ghat | Post | ORW031504051 | 790000 | 43000 |
| Ramghat | Pre | ORW031504037 | 192200 | 200 |
| Ramghat | Pre | ORW031504046 | 79000 | 1000 |
| Ramghat | During | ORW031504038 | 107000 | 2000 |
| Ramghat | During | ORW031504047 | 161000 | 1000 |
| Ramghat | Post | ORW031504039 | 110000 | 310 |
| Ramghat | Post | ORW031504048 | 128000 | 2000 |
| Kaliyadeh | Pre | ORW031504043 | 344000 | 1000 |
| Kaliyadeh | Pre | ORW031504052 | 139000 | 40 |
| Kaliyadeh | During | ORW031504044 | 153000 | 6000 |
| Kaliyadeh | During | ORW031504053 | 119000 | 4000 |
| Kaliyadeh | Post | ORW031504045 | 160000 | 1000 |
| Kaliyadeh | Post | ORW031504054 | 151000 | 1000 |
| Triveni Ghat | Pre | ORW041505058 | 25000 | 10000 |
| Triveni Ghat | Pre | ORW041505067 | 25600 | 1500 |
| Triveni Ghat | During | ORW041505059 | 80000 | 1000 |
| Triveni Ghat | During | ORW041505068 | 8000 | 2600 |
| Triveni Ghat | Post | ORW041505060 | 1300 | 10 |
| Triveni Ghat | Post | ORW041505069 | 3400 | 130 |
| Ramghat | Pre | ORW041505055 | 51800 | 1700 |
| Ramghat | Pre | ORW041505064 | 36000 | 2700 |
| Ramghat | During | ORW041505056 | 160000 | 52000 |
| Ramghat | During | ORW041505065 | 1680000 | 250000 |
| Ramghat | Post | ORW041505057 | 192000 | 4000 |
| Ramghat | Post | ORW041505066 | 192000 | 4000 |
| Kaliyadeh | Pre | ORW041505061 | 23600 | 3400 |
| Kaliyadeh | Pre | ORW041505070 | 52000 | 3000 |
| Kaliyadeh | During | ORW041505062 | 18900 | 2900 |
| Kaliyadeh | During | ORW041505071 | 12400 | 100 |
| Kaliyadeh | Post | ORW041505063 | 24000 | 2700 |
| Kaliyadeh | Post | ORW041505072 | 120000 | 3000 |
| Triveni Ghat | Pre | ORW051510076 | 22700 | 1100 |
| Triveni Ghat | Pre | ORW051510085 | 25500 | 2300 |
| Triveni Ghat | During | ORW051510077 | 2450 | 290 |
| Triveni Ghat | During | ORW051510086 | 3500 | 640 |
| Triveni Ghat | Post | ORW051510078 | 5290 | 2400 |
| Triveni Ghat | Post | ORW051510087 | 2560 | 420 |
| Ramghat | Pre | ORW051510073 | 15200 | 800 |
| Ramghat | Pre | ORW051510082 | 3650 | 710 |
| Ramghat | During | ORW051510074 | 3670 | 760 |
| Ramghat | During | ORW051510083 | 4060 | 680 |
| Ramghat | Post | ORW051510075 | 4250 | 1310 |
| Ramghat | Post | ORW051510084 | 215000 | 26000 |
| Kaliyadeh | Pre | ORW051510079 | 2720 | 20 |
| Kaliyadeh | Pre | ORW051510088 | 2630 | 50 |
| Kaliyadeh | During | ORW051510080 | 3260 | 220 |
| Kaliyadeh | During | ORW051510089 | 3230 | 350 |
| Kaliyadeh | Post | ORW051510081 | 3490 | 1170 |
| Kaliyadeh | Post | ORW051510090 | 2990 | 340 |
| Triveni Ghat | Pre | ORW061511094 | 1840 | 320 |
| Triveni Ghat | Pre | ORW061511103 | 3110 | 910 |
| Triveni Ghat | During | ORW061511095 | 2810 | 620 |
| Triveni Ghat | During | ORW061511104 | 4190 | 1480 |
| Triveni Ghat | Post | ORW061511096 | 2410 | 270 |
| Triveni Ghat | Post | ORW061511105 | 3040 | 110 |
| Ramghat | Pre | ORW061511091 | 1930 | 170 |
| Ramghat | Pre | ORW061511100 | 2620 | 290 |
| Ramghat | During | ORW061511092 | 3670 | 1350 |
| Ramghat | During | ORW061511101 | 3350 | 1230 |
| Ramghat | Post | ORW061511093 | 3110 | 760 |
| Ramghat | Post | ORW061511102 | 3110 | 910 |
| Kaliyadeh | Pre | ORW061511097 | 2280 | 190 |
| Kaliyadeh | Pre | ORW061511106 | 2640 | 40 |
| Kaliyadeh | During | ORW061511098 | 2460 | 380 |
| Kaliyadeh | During | ORW061511107 | 3270 | 670 |
| Kaliyadeh | Post | ORW061511099 | 2210 | 280 |
| Kaliyadeh | Post | ORW061511108 | 3290 | 270 |
| Triveni Ghat | Pre | ORW071601112 | 2130 | 410 |
| Triveni Ghat | Pre | ORW071601121 | 3080 | 150 |
| Triveni Ghat | During | ORW071601113 | 2770 | 150 |
| Triveni Ghat | During | ORW071601122 | 2230 | 70 |
| Triveni Ghat | Post | ORW071601114 | 2270 | 30 |
| Triveni Ghat | Post | ORW071601123 | 1280 | 120 |
| Ramghat | Pre | ORW071601109 | 2750 | 230 |
| Ramghat | Pre | ORW071601118 | 1730 | 40 |
| Ramghat | During | ORW071601110 | 2190 | 140 |
| Ramghat | During | ORW071601119 | 1830 | 560 |
| Ramghat | Post | ORW071601111 | 2660 | 70 |
| Ramghat | Post | ORW071601120 | 1890 | 200 |
| Kaliyadeh | Pre | ORW071601115 | 2650 | 10 |
| Kaliyadeh | Pre | ORW071601124 | 1830 | 70 |
| Kaliyadeh | During | ORW071601116 | 2650 | 410 |
| Kaliyadeh | During | ORW071601125 | 2200 | 220 |
| Kaliyadeh | Post | ORW071601117 | 1470 | 40 |
| Kaliyadeh | Post | ORW071601126 | 2200 | 220 |
| Triveni Ghat | Pre | ORW081602130 | 2060 | 20 |
| Triveni Ghat | Pre | ORW081602139 | 2530 | 50 |
| Triveni Ghat | During | ORW081602131 | 2100 | 180 |
| Triveni Ghat | During | ORW081602140 | 2340 | 180 |
| Triveni Ghat | Post | ORW081602132 | 2160 | 80 |
| Triveni Ghat | Post | ORW081602141 | 2610 | 90 |
| Ramghat | Pre | ORW081602127 | 1950 | 320 |
| Ramghat | Pre | ORW081602136 | 1680 | 160 |
| Ramghat | During | ORW081602128 | 2590 | 150 |
| Ramghat | During | ORW081602137 | 2790 | 200 |
| Ramghat | Post | ORW081602129 | 3320 | 1480 |
| Ramghat | Post | ORW081602138 | 3590 | 1720 |
| Kaliyadeh | Pre | ORW081602133 | 2200 | 350 |
| Kaliyadeh | Pre | ORW081602142 | 2190 | 50 |
| Kaliyadeh | During | ORW081602134 | 2780 | 1020 |
| Kaliyadeh | During | ORW081602143 | 2780 | 960 |
| Kaliyadeh | Post | ORW081602135 | 2710 | 720 |
| Kaliyadeh | Post | ORW081602144 | 2250 | 550 |

Table S5. Water quality of surface water during mass gathering in river Kshipra.

| **SPN** | **Period** | **Code** | **DOS** | Abtemt (oC) | Wtemp (oC) | **pH** | Cond (µS/cm) | **TDS (mg/l)** | **DO (mg/l)** | **Turb (NTU)** | **TSS (mg/l)** | **HCO3 Alk (mg/l)** | **Talk (mg/l)** | **Cl (mg/l)** | **TH (mg/l)** | **CaH (mg/l)** | **MgH (mg/l)** | **BOD (mg/l)** | **COD (mg/l)** | NO3-N (mg/l) | **TP (mg/l)** | **OrthoP (mg/l)** | **OrgP (mg/l)** |
| --- | --- | --- | --- | --- | --- | --- | --- | --- | --- | --- | --- | --- | --- | --- | --- | --- | --- | --- | --- | --- | --- | --- | --- |
| Triveni Ghat | Pre | ORW011408004 | 24/08/2014 | 35 | 32 | 9.7 | 793 | 470 | 13.2 | 7.8 | 18 | 164 | 204 | 97.902 | 256.2 | 128 | 128.2 | 32 | 88 | 13.8 | 6.32 | 4.05 | 2.27 |
| Triveni Ghat | Pre | ORW011408013 | 24/08/2014 | 35 | 32 | 9.7 | 830 | 470 | 13.6 | 8.5 | 19 | 144 | 182 | 94.905 | 245.7 | 138 | 107.7 | 36 | 104 | 14.7 | 5.89 | 4.63 | 1.26 |
| Triveni Ghat | During | ORW011408005 | 25/08/2014 | 30 | 29 | 8.7 | 1040 | 650 | 6.8 | 13.1 | 24 | 190 | 206 | 123.876 | 304.5 | 170 | 134.5 | 48 | 120 | 5.4 | 5.11 | 4.76 | 0.35 |
| Triveni Ghat | During | ORW011408014 | 25/08/2014 | 30 | 29 | 8.6 | 1084 | 670 | 6.8 | 13.3 | 23 | 202 | 218 | 126.873 | 306.6 | 168 | 138.6 | 32 | 104 | 4.6 | 4.68 | 4.41 | 0.27 |
| Triveni Ghat | Post | ORW011408006 | 25/08/2014 | 34 | 31 | 8.6 | 1163 | 710 | 9.6 | 14.2 | 24.5 | 236 | 248 | 137.862 | 338.1 | 186 | 152.1 | 32 | 88 | 11.7 | 8.76 | 7.02 | 1.74 |
| Triveni Ghat | Post | ORW011408015 | 25/08/2014 | 34 | 31 | 8.6 | 1187 | 730 | 10.4 | 11.8 | 21.5 | 232 | 246 | 141.858 | 352.8 | 196 | 156.8 | 32 | 96 | 10.2 | 8.03 | 7.56 | 0.47 |
| Ramghat | Pre | ORW011408001 | 24/08/2014 | 32 | 30 | 8.2 | 1071 | 660 | 4.4 | 20.5 | 32 | 198 | 200 | 125.874 | 308.7 | 180 | 128.7 | 28 | 80 | 6.9 | 5.67 | 4.56 | 1.11 |
| Ramghat | Pre | ORW011408010 | 24/08/2014 | 32 | 30 | 8.3 | 1109 | 670 | 3.6 | 21.4 | 36 | 198 | 202 | 121.878 | 329.7 | 184 | 145.7 | 24 | 64 | 6.4 | 5.12 | 4.89 | 0.23 |
| Ramghat | During | ORW011408002 | 25/08/2014 | 35 | 31 | 7.7 | 1193 | 710 | 3.2 | 34.5 | 41 | 228 | 228 | 134.865 | 346.5 | 200 | 146.5 | 88 | 240 | 8.3 | 7.19 | 6.75 | 0.44 |
| Ramghat | During | ORW011408011 | 25/08/2014 | 35 | 31 | 8 | 1198 | 710 | 3.6 | 36.1 | 42.5 | 230 | 230 | 137.862 | 346.5 | 200 | 146.5 | 64 | 192 | 8.2 | 7.56 | 5.76 | 1.8 |
| Ramghat | Post | ORW011408003 | 25/08/2014 | 31 | 32 | 8.2 | 1177 | 750 | 2.4 | 98.7 | 51 | 240 | 240 | 145.854 | 348.6 | 222 | 126.6 | 64 | 208 | 20.5 | 9.12 | 6.18 | 2.94 |
| Ramghat | Post | ORW011408012 | 25/08/2014 | 31 | 32 | 8.2 | 1232 | 750 | 2.4 | 89.1 | 45.5 | 234 | 234 | 142.857 | 357 | 206 | 151 | 48 | 136 | 16.1 | 10.24 | 7.1 | 3.14 |
| Kaliyadeh | Pre | ORW011408007 | 24/08/2014 | 30 | 28 | 8.1 | 1187 | 760 | 3.2 | 7.4 | 17 | 232 | 232 | 144.855 | 373.8 | 202 | 171.8 | 16 | 48 | 5.4 | 4.98 | 4.34 | 0.64 |
| Kaliyadeh | Pre | ORW011408016 | 24/08/2014 | 30 | 28 | 8.2 | 1220 | 760 | 3.2 | 6.9 | 14.5 | 220 | 220 | 143.856 | 367.5 | 206 | 161.5 | 16 | 40 | 4.4 | 4.15 | 3.56 | 0.59 |
| Kaliyadeh | During | ORW011408008 | 25/08/2014 | 35 | 32 | 8.2 | 1237 | 760 | 3.2 | 12.2 | 17 | 242 | 242 | 151.848 | 394.8 | 210 | 184.8 | 24 | 64 | 14.9 | 5.36 | 3.33 | 2.03 |
| Kaliyadeh | During | ORW011408017 | 25/08/2014 | 35 | 32 | 8.2 | 1284 | 780 | 3.2 | 17.1 | 31.5 | 228 | 228 | 154.845 | 399 | 226 | 173 | 24 | 64 | 16.1 | 5.11 | 3.47 | 1.64 |
| Kaliyadeh | Post | ORW011408009 | 25/08/2014 | 30 | 29 | 8.2 | 1251 | 790 | 2.4 | 8.1 | 21 | 226 | 226 | 148.851 | 354.9 | 202 | 152.9 | 40 | 112 | 11.3 | 8.87 | 7.12 | 1.75 |
| Kaliyadeh | Post | ORW011408018 | 25/08/2014 | 30 | 29 | 8.2 | 1247 | 780 | 2.8 | 5.8 | 18.5 | 238 | 238 | 147.852 | 350.7 | 206 | 144.7 | 24 | 64 | 12.5 | 7.78 | 6.85 | 0.93 |
| Triveni Ghat | Pre | ORW021411022 | 21/11/2014 | 29 | 26 | 8.5 | 1337 | 800 | 4.6 | 22 | 37.5 | 312 | 318 | 183.816 | 414 | 233.1 | 180.9 | 24 | 68 | 5.84 | 4.78 | 3.78 | 1 |
| Triveni Ghat | Pre | ORW021411031 | 21/11/2014 | 29 | 26 | 8.5 | 1226 | 790 | 4.6 | 23.4 | 32.5 | 306 | 312 | 188.811 | 424 | 231 | 193 | 24 | 72 | 5.94 | 4.81 | 3.82 | 0.99 |
| Triveni Ghat | During | ORW021411023 | 22/11/2014 | 27 | 28 | 8.4 | 1336 | 780 | 4.4 | 24.4 | 83.5 | 310 | 310 | 191.808 | 428 | 235.2 | 192.8 | 88 | 264 | 5.92 | 4.92 | 3.99 | 0.93 |
| Triveni Ghat | During | ORW021411032 | 22/11/2014 | 27 | 28 | 8.4 | 1215 | 780 | 4 | 22.9 | 80 | 316 | 316 | 184.815 | 400 | 241.5 | 158.5 | 88 | 240 | 5.85 | 4.85 | 4.03 | 0.82 |
| Triveni Ghat | Post | ORW021411024 | 22/11/2014 | 30 | 26 | 8.8 | 1334 | 780 | 6.4 | 84.2 | 119.5 | 336 | 344 | 189.81 | 394 | 228.9 | 165.1 | 88 | 240 | 5.89 | 4.86 | 3.82 | 1.04 |
| Triveni Ghat | Post | ORW021411033 | 22/11/2014 | 30 | 26 | 8.8 | 1184 | 780 | 6.4 | 67.8 | 117.5 | 328 | 338 | 186.813 | 398 | 228.9 | 169.1 | 72 | 200 | 5.74 | 4.73 | 3.74 | 0.99 |
| Ramghat | Pre | ORW021411019 | 21/11/2014 | 30 | 27 | 9.8 | 1546 | 880 | 13.2 | 37.3 | 48 | 366 | 418 | 221.778 | 442 | 222.6 | 219.4 | 16 | 40 | 5.64 | 4.44 | 3.62 | 0.82 |
| Ramghat | Pre | ORW021411028 | 21/11/2014 | 30 | 27 | 9.8 | 1488 | 880 | 14 | 37.8 | 60 | 358 | 410 | 234.765 | 454 | 220.5 | 233.5 | 24 | 64 | 5.58 | 4.51 | 3.59 | 0.92 |
| Ramghat | During | ORW021411020 | 22/11/2014 | 28 | 29 | 9.3 | 1534 | 880 | 8 | 33.7 | 44.5 | 356 | 372 | 233.766 | 464 | 262.5 | 201.5 | 48 | 136 | 6.62 | 4.56 | 3.72 | 0.84 |
| Ramghat | During | ORW021411029 | 22/11/2014 | 28 | 29 | 9.4 | 1368 | 890 | 7.6 | 38.5 | 52.5 | 368 | 382 | 236.763 | 450 | 270.9 | 179.1 | 64 | 172 | 6.58 | 4.62 | 3.78 | 0.84 |
| Ramghat | Post | ORW021411021 | 22/11/2014 | 30 | 28 | 9.3 | 1556 | 880 | 9.8 | 41.5 | 69.5 | 362 | 378 | 231.768 | 450 | 235.2 | 214.8 | 48 | 144 | 5.76 | 4.48 | 3.64 | 0.84 |
| Ramghat | Post | ORW021411030 | 22/11/2014 | 30 | 25 | 9.3 | 1467 | 870 | 9 | 43.8 | 85 | 368 | 386 | 231.768 | 460 | 262.5 | 197.5 | 48 | 136 | 5.81 | 4.56 | 3.72 | 0.84 |
| Kaliyadeh | Pre | ORW021411025 | 21/11/2014 | 31 | 25 | 8.6 | 1356 | 780 | 5.08 | 9.4 | 29 | 332 | 336 | 191.808 | 394 | 235.2 | 158.8 | 16 | 48 | 4.42 | 3.71 | 2.86 | 0.85 |
| Kaliyadeh | Pre | ORW021411034 | 21/11/2014 | 31 | 25 | 8.7 | 1197 | 780 | 5.4 | 7.3 | 25 | 330 | 336 | 186.813 | 408 | 256.2 | 151.8 | 20 | 64 | 4.39 | 3.69 | 2.82 | 0.87 |
| Kaliyadeh | During | ORW021411026 | 22/11/2014 | 31 | 23 | 8.6 | 1357 | 800 | 4.8 | 13.45 | 38 | 330 | 336 | 193.806 | 416 | 218.4 | 197.6 | 24 | 80 | 4.39 | 3.82 | 2.42 | 1.4 |
| Kaliyadeh | During | ORW021411035 | 22/11/2014 | 31 | 23 | 8.6 | 1174 | 810 | 5 | 9.8 | 29.5 | 330 | 336 | 192.807 | 412 | 239.4 | 172.6 | 28 | 80 | 4.41 | 3.86 | 2.51 | 1.35 |
| Kaliyadeh | Post | ORW021411027 | 22/11/2014 | 29 | 23 | 8.6 | 1354 | 790 | 5 | 13.62 | 43 | 326 | 334 | 188.811 | 412 | 218.4 | 193.6 | 32 | 88 | 4.33 | 3.64 | 2.66 | 0.98 |
| Kaliyadeh | Post | ORW021411036 | 22/11/2014 | 29 | 23 | 8.6 | 1297 | 790 | 5.2 | 12.95 | 39 | 338 | 346 | 190.809 | 406 | 224.7 | 181.3 | 32 | 96 | 4.39 | 3.54 | 2.71 | 0.83 |
| Triveni Ghat | Pre | ORW031504040 | 17/04/2015 | 33 | 32 | 8.4 | 840 | 681 | 3.6 | 16.22 | 33 | 428 | 456 | 146.853 | 336 | 216.3 | 119.7 | 32 | 68 | 9.562 | 2.468 | 1.782 | 0.686 |
| Triveni Ghat | Pre | ORW031504049 | 17/04/2015 | 33 | 32 | 8.4 | 836 | 680 | 2.8 | 9.73 | 25 | 416 | 444 | 181.818 | 260 | 117.2 | 142.8 | 36 | 72 | 9.462 | 2.345 | 1.722 | 0.623 |
| Triveni Ghat | During | ORW031504041 | 18/04/2015 | 30 | 23 | 7.9 | 830 | 680 | 10.8 | 14.95 | 68.5 | 426 | 426 | 179.82 | 326 | 202.1 | 123.9 | 80 | 192 | 10.012 | 2.523 | 1.702 | 0.821 |
| Triveni Ghat | During | ORW031504050 | 18/04/2015 | 30 | 23 | 7.9 | 822 | 678 | 10.4 | 13.84 | 60.5 | 440 | 440 | 173.826 | 348 | 217.8 | 130.2 | 88 | 196 | 9.568 | 2.521 | 1.658 | 0.863 |
| Triveni Ghat | Post | ORW031504042 | 18/04/2015 | 33 | 25 | 7.7 | 930 | 712 | Nil | 144.5 | 112.5 | 446 | 446 | 187.812 | 316 | 202.6 | 113.4 | 96 | 216 | 9.228 | 5.462 | 2.324 | 3.138 |
| Triveni Ghat | Post | ORW031504051 | 18/04/2015 | 33 | 25 | 7.6 | 925 | 710 | Nil | 103.9 | 98 | 460 | 460 | 199.8 | 308 | 211.4 | 96.6 | 112 | 220 | 9.452 | 5.566 | 2.442 | 3.124 |
| Ramghat | Pre | ORW031504037 | 17/04/2015 | 31 | 29 | 8.5 | 740 | 612 | 28.8 | 22 | 36.5 | 376 | 386 | 178.821 | 238 | 160.3 | 77.7 | 32 | 64 | 10.254 | 3.264 | 1.472 | 1.792 |
| Ramghat | Pre | ORW031504046 | 17/04/2015 | 31 | 29 | 8.5 | 755 | 617 | 27.2 | 28.1 | 45 | 374 | 392 | 171.828 | 296 | 222.5 | 73.5 | 28 | 60 | 10.326 | 3.336 | 1.425 | 1.911 |
| Ramghat | During | ORW031504038 | 18/04/2015 | 32 | 26 | 7.6 | 820 | 650 | 7.2 | 18.4 | 52.5 | 378 | 378 | 179.82 | 304 | 184.3 | 119.7 | 40 | 72 | 11.426 | 3.346 | 1.354 | 1.992 |
| Ramghat | During | ORW031504047 | 18/04/2015 | 32 | 26 | 7.6 | 835 | 650 | 6.8 | 20.8 | 56.5 | 394 | 394 | 166.833 | 328 | 197.8 | 130.2 | 40 | 88 | 11.412 | 3.412 | 1.456 | 1.956 |
| Ramghat | Post | ORW031504039 | 18/04/2015 | 36 | 29 | 8.4 | 810 | 630 | 36 | 25.2 | 71 | 410 | 438 | 170.829 | 334 | 184.9 | 149.1 | 48 | 96 | 11.442 | 3.446 | 1.421 | 2.025 |
| Ramghat | Post | ORW031504048 | 18/04/2015 | 36 | 29 | 8.5 | 845 | 670 | 35.2 | 36.4 | 84.5 | 390 | 420 | 181.818 | 336 | 193.2 | 142.8 | 48 | 92 | 10.356 | 3.562 | 1.357 | 2.205 |
| Kaliyadeh | Pre | ORW031504043 | 17/04/2015 | 31 | 28 | 7.9 | 1080 | 826 | 24.8 | 12.28 | 26.5 | 390 | 394 | 183.816 | 270 | 186 | 84 | 20 | 56 | 7.322 | 3.056 | 2.567 | 0.489 |
| Kaliyadeh | Pre | ORW031504052 | 17/04/2015 | 32 | 28 | 7.7 | 1102 | 852 | 24.4 | 11.82 | 20 | 394 | 398 | 177.822 | 272 | 185.9 | 86.1 | 16 | 48 | 7.356 | 3.063 | 2.552 | 0.511 |
| Kaliyadeh | During | ORW031504044 | 18/04/2015 | 33 | 25 | 7.7 | 845 | 646 | 9.2 | 14.68 | 19 | 404 | 404 | 186.813 | 342 | 201.3 | 140.7 | 24 | 64 | 7.456 | 3.156 | 2.124 | 1.032 |
| Kaliyadeh | During | ORW031504053 | 18/04/2015 | 33 | 25 | 7.8 | 852 | 657 | 8.8 | 16.9 | 29.5 | 392 | 392 | 184.815 | 314 | 206.9 | 107.1 | 24 | 60 | 7.265 | 3.246 | 2.635 | 0.611 |
| Kaliyadeh | Post | ORW031504045 | 18/04/2015 | 36 | 29 | 8.2 | 835 | 630 | 37.2 | 19.8 | 35 | 386 | 400 | 184.815 | 260 | 180.2 | 79.8 | 32 | 76 | 8.213 | 3.887 | 2.725 | 1.162 |
| Kaliyadeh | Post | ORW031504054 | 18/04/2015 | 36 | 29 | 8.3 | 829 | 629 | 36.8 | 18.8 | 30 | 370 | 382 | 197.802 | 370 | 210.4 | 159.6 | 24 | 56 | 7.869 | 3.223 | 2.426 | 0.797 |
| Triveni Ghat | Pre | ORW041505058 | 17/05/2015 | 43 | 35 | 8.4 | 1115 | 800 | 3.2 | 23.12 | 97 | 408 | 411 | 134.865 | 316 | 194.2 | 121.8 | 52 | 144 | 12.346 | 3.126 | 0.895 | 2.231 |
| Triveni Ghat | Pre | ORW041505067 | 17/05/2015 | 43 | 35 | 8.4 | 1080 | 790 | 3.2 | 21.21 | 89 | 414 | 417 | 175.824 | 284 | 170.6 | 113.4 | 56 | 140 | 12.224 | 3.124 | 1.018 | 2.106 |
| Triveni Ghat | During | ORW041505059 | 18/05/2015 | 32 | 30 | 8.2 | 940 | 721 | 2 | 12.23 | 54.5 | 436 | 436 | 177.822 | 328 | 218.8 | 109.2 | 64 | 156 | 11.256 | 3.689 | 1.066 | 2.623 |
| Triveni Ghat | During | ORW041505068 | 18/05/2015 | 32 | 30 | 8.3 | 962 | 742 | 2 | 14.35 | 64 | 434 | 434 | 175.824 | 336 | 205.8 | 130.2 | 72 | 160 | 11.568 | 3.524 | 1.158 | 2.366 |
| Triveni Ghat | Post | ORW041505060 | 18/05/2015 | 43 | 34 | 8.2 | 911 | 670 | 1.2 | 20.8 | 104 | 458 | 458 | 191.808 | 296 | 184.7 | 111.3 | 64 | 152 | 11.218 | 3.362 | 2.354 | 1.008 |
| Triveni Ghat | Post | ORW041505069 | 18/05/2015 | 43 | 34 | 8.3 | 925 | 680 | 1.6 | 20.3 | 88 | 456 | 456 | 195.804 | 310 | 211.3 | 98.7 | 56 | 148 | 11.422 | 3.526 | 2.142 | 1.384 |
| Ramghat | Pre | ORW041505055 | 17/05/2015 | 43 | 34 | 9.3 | 985 | 780 | 19.2 | 19.2 | 50 | 350 | 382 | 162.837 | 256 | 167.8 | 88.2 | 44 | 108 | 12.816 | 3.568 | 1.827 | 1.741 |
| Ramghat | Pre | ORW041505064 | 17/05/2015 | 43 | 34 | 9.3 | 993 | 780 | 19.2 | 18.7 | 47.5 | 356 | 386 | 164.835 | 272 | 183.8 | 88.2 | 40 | 96 | 12.266 | 3.569 | 1.845 | 1.724 |
| Ramghat | During | ORW041505056 | 18/05/2015 | 30 | 29 | 8.8 | 934 | 715 | 6 | 35.6 | 65 | 372 | 386 | 181.818 | 284 | 170.6 | 113.4 | 40 | 92 | 12.526 | 3.886 | 2.144 | 1.742 |
| Ramghat | During | ORW041505065 | 18/05/2015 | 30 | 29 | 8.8 | 918 | 714 | 6.4 | 42.6 | 75.5 | 382 | 396 | 183.816 | 318 | 202.5 | 115.5 | 32 | 84 | 12.635 | 3.826 | 2.156 | 1.67 |
| Ramghat | Post | ORW041505057 | 18/05/2015 | 44 | 34 | 9.1 | 908 | 652 | 7.6 | 30.6 | 54 | 396 | 424 | 171.828 | 336 | 182.7 | 153.3 | 80 | 212 | 13.894 | 5.366 | 2.422 | 2.944 |
| Ramghat | Post | ORW041505066 | 18/05/2015 | 44 | 34 | 9.2 | 925 | 666 | 6.8 | 31.4 | 59.5 | 392 | 424 | 175.824 | 344 | 194.9 | 149.1 | 80 | 220 | 13.358 | 5.335 | 2.457 | 2.878 |
| Kaliyadeh | Pre | ORW041505061 | 17/05/2015 | 44 | 33 | 8.7 | 958 | 693 | 8.4 | 12.28 | 42.5 | 406 | 416 | 186.813 | 272 | 183.8 | 88.2 | 52 | 112 | 8.122 | 2.948 | 1.225 | 1.723 |
| Kaliyadeh | Pre | ORW041505070 | 17/05/2015 | 44 | 33 | 8.7 | 961 | 694 | 8 | 15.36 | 47.5 | 412 | 422 | 181.818 | 284 | 195.8 | 88.2 | 48 | 92 | 8.135 | 2.865 | 1.218 | 1.647 |
| Kaliyadeh | During | ORW041505062 | 18/05/2015 | 35 | 31 | 8.2 | 942 | 715 | 1.6 | 19.42 | 59.5 | 436 | 436 | 194.805 | 372 | 281.7 | 90.3 | 56 | 120 | 8.246 | 2.756 | 1.338 | 1.418 |
| Kaliyadeh | During | ORW041505071 | 18/05/2015 | 35 | 31 | 8.2 | 931 | 680 | 2 | 17.93 | 62.5 | 432 | 432 | 195.804 | 324 | 229.5 | 94.5 | 56 | 124 | 8.212 | 2.822 | 1.356 | 1.466 |
| Kaliyadeh | Post | ORW041505063 | 18/05/2015 | 44 | 33 | 8.4 | 915 | 666 | 2.8 | 18.63 | 48.5 | 390 | 396 | 175.824 | 284 | 195.8 | 88.2 | 48 | 116 | 8.167 | 3.106 | 1.825 | 1.281 |
| Kaliyadeh | Post | ORW041505072 | 18/05/2015 | 44 | 33 | 8.4 | 906 | 662 | 3.2 | 18.35 | 47.5 | 386 | 390 | 178.821 | 336 | 256.2 | 79.8 | 56 | 128 | 8.182 | 3.085 | 1.766 | 1.319 |
| Triveni Ghat | Pre | ORW051510076 | 11/10/2015 | 36 | 31 | 8.6 | 1187 | 840 | 5.2 | 24.5 | 28.5 | 406 | 420 | 217.782 | 388 | 203.2 | 184.8 | 18 | 48 | 5.242 | 3.482 | 3.78 | -0.298 |
| Triveni Ghat | Pre | ORW051510085 | 11/10/2015 | 36 | 31 | 8.6 | 1192 | 840 | 5.6 | 24.8 | 22.5 | 408 | 424 | 214.785 | 390 | 217.8 | 172.2 | 16 | 40 | 5.125 | 3.625 | 3.82 | -0.195 |
| Triveni Ghat | During | ORW051510077 | 12/10/2015 | 35 | 30 | 8.4 | 1222 | 880 | 5.2 | 26.4 | 35 | 396 | 402 | 215.784 | 276 | 185.7 | 90.3 | 22 | 56 | 5.336 | 3.55 | 3.99 | -0.44 |
| Triveni Ghat | During | ORW051510086 | 12/10/2015 | 35 | 30 | 8.4 | 1215 | 870 | 4.8 | 23.3 | 38.5 | 396 | 402 | 217.782 | 272 | 175.4 | 96.6 | 22 | 60 | 5.364 | 3.635 | 4.03 | -0.395 |
| Triveni Ghat | Post | ORW051510078 | 12/10/2015 | 35 | 29 | 8.5 | 1235 | 880 | 4.4 | 18.6 | 40 | 388 | 404 | 222.777 | 392 | 163.1 | 228.9 | 24 | 72 | 5.224 | 3.214 | 3.82 | -0.606 |
| Triveni Ghat | Post | ORW051510087 | 12/10/2015 | 35 | 29 | 8.5 | 1232 | 880 | 4.8 | 19.3 | 43.5 | 384 | 398 | 224.775 | 394 | 173.5 | 220.5 | 26 | 76 | 5.458 | 3.218 | 3.74 | -0.522 |
| Ramghat | Pre | ORW051510073 | 11/10/2015 | 35 | 30 | 8.7 | 1080 | 820 | 6.4 | 42.6 | 32.5 | 400 | 410 | 218.781 | 304 | 173.8 | 130.2 | 12 | 32 | 5.826 | 4.56 | 2.22 | 2.34 |
| Ramghat | Pre | ORW051510082 | 11/10/2015 | 35 | 30 | 8.6 | 1105 | 820 | 7.2 | 42.8 | 44 | 404 | 414 | 217.782 | 306 | 173.7 | 132.3 | 14 | 40 | 5.861 | 4.453 | 2.241 | 2.212 |
| Ramghat | During | ORW051510074 | 12/10/2015 | 36 | 31 | 8.6 | 1180 | 860 | 7.6 | 30.6 | 56 | 404 | 422 | 211.788 | 396 | 177.6 | 218.4 | 30 | 88 | 6.24 | 4.634 | 2.168 | 2.466 |
| Ramghat | During | ORW051510083 | 12/10/2015 | 36 | 31 | 8.6 | 1175 | 860 | 7.6 | 32.4 | 63 | 406 | 422 | 211.788 | 392 | 169.4 | 222.6 | 34 | 96 | 6.58 | 4.612 | 2.157 | 2.455 |
| Ramghat | Post | ORW051510075 | 12/10/2015 | 35 | 30 | 8.4 | 1207 | 860 | 4.4 | 40.6 | 78 | 416 | 428 | 224.775 | 362 | 101.6 | 260.4 | 34 | 96 | 6.662 | 4.619 | 2.824 | 1.795 |
| Ramghat | Post | ORW051510084 | 12/10/2015 | 35 | 30 | 8.4 | 1210 | 870 | 4.8 | 40.3 | 85.5 | 412 | 422 | 218.781 | 368 | 111.8 | 256.2 | 34 | 112 | 6.815 | 4.624 | 2.786 | 1.838 |
| Kaliyadeh | Pre | ORW051510079 | 11/10/2015 | 35 | 29 | 8.3 | 1043 | 800 | 4.8 | 18.9 | 43.5 | 364 | 368 | 222.777 | 246 | 168.3 | 77.7 | 18 | 56 | 4.335 | 3.124 | 2.108 | 1.016 |
| Kaliyadeh | Pre | ORW051510088 | 11/10/2015 | 35 | 29 | 8.4 | 1055 | 800 | 4.4 | 19.6 | 48 | 372 | 376 | 220.779 | 244 | 168.4 | 75.6 | 16 | 40 | 4.34 | 3.122 | 2.102 | 1.02 |
| Kaliyadeh | During | ORW051510080 | 12/10/2015 | 33 | 28 | 8.4 | 1142 | 840 | 5.2 | 60.53 | 71 | 366 | 366 | 199.8 | 350 | 131.6 | 218.4 | 16 | 36 | 4.398 | 4.145 | 2.226 | 1.919 |
| Kaliyadeh | During | ORW051510089 | 12/10/2015 | 33 | 28 | 8.3 | 1170 | 850 | 4.8 | 60.23 | 79 | 368 | 368 | 202.797 | 358 | 152.2 | 205.8 | 16 | 40 | 4.685 | 4.175 | 2.45 | 1.725 |
| Kaliyadeh | Post | ORW051510081 | 12/10/2015 | 34 | 29 | 8.4 | 1192 | 870 | 5.2 | 52.8 | 68 | 386 | 396 | 127.872 | 364 | 200.2 | 163.8 | 18 | 48 | 4.658 | 4.122 | 2.358 | 1.764 |
| Kaliyadeh | Post | ORW051510090 | 12/10/2015 | 34 | 29 | 8,4 | 1215 | 890 | 4.8 | 68.66 | 77 | 388 | 398 | 219.78 | 370 | 214.6 | 155.4 | 20 | 56 | 4.645 | 4.268 | 2.486 | 1.782 |
| Triveni Ghat | Pre | ORW061511094 | 24/11/2015 | 29 | 26 | 8.7 | 1010 | 670 | 10.8 | 28.56 | 54.5 | 372 | 388 | 187.812 | 478 | 223.9 | 254.1 | 20 | 52 | 6.312 | 3.856 | 1.362 | 2.494 |
| Triveni Ghat | Pre | ORW061511103 | 24/11/2015 | 29 | 26 | 8.7 | 1023 | 710 | 10.4 | 27.86 | 40.5 | 370 | 388 | 189.81 | 474 | 219.9 | 254.1 | 20 | 60 | 6.328 | 3.842 | 1.355 | 2.487 |
| Triveni Ghat | During | ORW061511095 | 25/11/2015 | 26 | 24 | 8.5 | 970 | 686 | 4.4 | 22.36 | 69.5 | 370 | 382 | 191.808 | 474 | 253.5 | 220.5 | 24 | 68 | 6.326 | 3.894 | 2.022 | 1.872 |
| Triveni Ghat | During | ORW061511104 | 25/11/2015 | 26 | 24 | 8.5 | 1012 | 710 | 4 | 22.66 | 60 | 358 | 368 | 187.812 | 468 | 251.7 | 216.3 | 26 | 68 | 6.314 | 4.202 | 2.009 | 2.193 |
| Triveni Ghat | Post | ORW061511096 | 25/11/2015 | 30 | 26 | 8.7 | 1024 | 720 | 9.2 | 21.56 | 58.5 | 364 | 390 | 190.809 | 456 | 229.2 | 226.8 | 26 | 64 | 6.106 | 4.12 | 2.186 | 1.934 |
| Triveni Ghat | Post | ORW061511105 | 25/11/2015 | 30 | 26 | 8.7 | 985 | 710 | 8.8 | 23.82 | 68.5 | 362 | 384 | 195.804 | 466 | 232.9 | 233.1 | 26 | 76 | 6.118 | 4.116 | 2.154 | 1.962 |
| Ramghat | Pre | ORW061511091 | 24/11/2015 | 30 | 26 | 8.8 | 1060 | 780 | 11.2 | 33.6 | 54 | 358 | 376 | 234.765 | 502 | 283.6 | 218.4 | 18 | 48 | 5.535 | 3.895 | 2.246 | 1.649 |
| Ramghat | Pre | ORW061511100 | 24/11/2015 | 30 | 26 | 8.8 | 1042 | 780 | 10.8 | 40.63 | 66.5 | 356 | 374 | 232.767 | 518 | 299.6 | 218.4 | 24 | 60 | 5.546 | 3.786 | 2.208 | 1.578 |
| Ramghat | During | ORW061511092 | 25/11/2015 | 28 | 24 | 8.5 | 1086 | 820 | 6.4 | 66.22 | 96.5 | 366 | 384 | 239.76 | 508 | 293.8 | 214.2 | 32 | 88 | 7.558 | 5.046 | 2.386 | 2.66 |
| Ramghat | During | ORW061511101 | 25/11/2015 | 28 | 24 | 8.5 | 1126 | 830 | 6.8 | 65.25 | 90.5 | 344 | 360 | 218.781 | 484 | 267.7 | 216.3 | 34 | 100 | 7.515 | 5.038 | 2.412 | 2.626 |
| Ramghat | Post | ORW061511093 | 25/11/2015 | 31 | 24 | 8.5 | 1096 | 810 | 4.8 | 55.21 | 86.5 | 352 | 364 | 226.773 | 486 | 278.1 | 207.9 | 34 | 96 | 7.634 | 5.128 | 2.558 | 2.57 |
| Ramghat | Post | ORW061511102 | 25/11/2015 | 31 | 24 | 8.5 | 1082 | 810 | 5.6 | 52.65 | 83 | 348 | 362 | 224.775 | 492 | 294.6 | 197.4 | 34 | 104 | 7.628 | 5.132 | 2.719 | 2.413 |
| Kaliyadeh | Pre | ORW061511097 | 24/11/2015 | 29 | 25 | 8.8 | 1022 | 760 | 5.2 | 17.65 | 34.5 | 404 | 434 | 225.774 | 486 | 259.2 | 226.8 | 18 | 40 | 4.85 | 3.062 | 2.002 | 1.06 |
| Kaliyadeh | Pre | ORW061511106 | 24/11/2015 | 29 | 25 | 8.8 | 1027 | 770 | 6 | 22.45 | 44.5 | 396 | 428 | 196.803 | 494 | 265.1 | 228.9 | 20 | 48 | 4.856 | 3.102 | 1.869 | 1.233 |
| Kaliyadeh | During | ORW061511098 | 25/11/2015 | 28 | 24 | 8.6 | 1076 | 820 | 4 | 22.5 | 23.5 | 416 | 428 | 197.802 | 484 | 250.9 | 233.1 | 20 | 52 | 4.592 | 3.122 | 2.109 | 1.013 |
| Kaliyadeh | During | ORW061511107 | 25/11/2015 | 28 | 24 | 8.6 | 1069 | 820 | 4.4 | 22.76 | 41.5 | 412 | 424 | 192.807 | 456 | 227.1 | 228.9 | 20 | 56 | 4.786 | 3.128 | 2.115 | 1.013 |
| Kaliyadeh | Post | ORW061511099 | 25/11/2015 | 30 | 26 | 8.6 | 1033 | 780 | 5.6 | 25.46 | 49.5 | 384 | 412 | 201.798 | 476 | 272.3 | 203.7 | 22 | 64 | 4.887 | 3.224 | 2.124 | 1.1 |
| Kaliyadeh | Post | ORW061511108 | 25/11/2015 | 30 | 26 | 8.6 | 1040 | 780 | 6 | 24.86 | 53 | 388 | 414 | 198.801 | 484 | 276.1 | 207.9 | 22 | 60 | 4.896 | 3.246 | 2.13 | 1.116 |
| Triveni Ghat | Pre | ORW071601112 | 08/01/2016 | 26 | 24 | 7.7 | 1123 | 810 | 5.6 | 462 | 50.2 | 292 | 292 | 186.813 | 410 | 288.2 | 121.8 | 9.6 | 76 | 6.995 | 3.245 | 0.98 | 2.265 |
| Triveni Ghat | Pre | ORW071601121 | 08/01/2016 | 26 | 24 | 7.7 | 1145 | 800 | 5.2 | 372 | 48.2 | 354 | 354 | 181.818 | 424 | 283.3 | 140.7 | 8 | 88 | 7.235 | 3.264 | 1.699 | 1.565 |
| Triveni Ghat | During | ORW071601113 | 09/01/2016 | 19 | 22 | 8.2 | 971 | 710 | 6.4 | 35.8 | 22.3 | 242 | 246 | 168.831 | 364 | 259 | 105 | 9.2 | 36 | 3.121 | 0.812 | 0.232 | 0.58 |
| Triveni Ghat | During | ORW071601122 | 09/01/2016 | 19 | 22 | 8.1 | 960 | 700 | 6.8 | 37.2 | 24.8 | 250 | 254 | 168.831 | 368 | 235.7 | 132.3 | 10 | 48 | 3.129 | 0.756 | 0.374 | 0.382 |
| Triveni Ghat | Post | ORW071601114 | 09/01/2016 | 26 | 20 | 8.2 | 926 | 700 | 7.2 | 31.2 | 8.3 | 230 | 240 | 180.819 | 358 | 246.7 | 111.3 | 9.6 | 48 | 3.452 | 0.668 | 0.445 | 0.223 |
| Triveni Ghat | Post | ORW071601123 | 09/01/2016 | 26 | 20 | 8.1 | 915 | 710 | 7.2 | 27.8 | 9.7 | 214 | 220 | 175.824 | 364 | 259 | 105 | 13.6 | 36 | 3.569 | 0.598 | 0.247 | 0.351 |
| Ramghat | Pre | ORW071601109 | 08/01/2016 | 28 | 24 | 8.3 | 1247 | 940 | 12.4 | 39.9 | 14.3 | 358 | 386 | 215.784 | 432 | 360.6 | 71.4 | 26.4 | 76 | 4.663 | 1.989 | 1.942 | 0.047 |
| Ramghat | Pre | ORW071601118 | 08/01/2016 | 28 | 24 | 8.4 | 1235 | 940 | 12.8 | 43.3 | 15.9 | 342 | 370 | 227.772 | 508 | 274.9 | 233.1 | 28.4 | 100 | 5.289 | 1.142 | 1.128 | 0.014 |
| Ramghat | During | ORW071601110 | 09/01/2016 | 21 | 22 | 7.8 | 1217 | 930 | 5.2 | 37.4 | 20.1 | 360 | 360 | 230.769 | 488 | 240.2 | 247.8 | 16.4 | 68 | 5.524 | 2.766 | 2.154 | 0.612 |
| Ramghat | During | ORW071601119 | 09/01/2016 | 21 | 22 | 7.8 | 1260 | 950 | 4.8 | 62.6 | 29.1 | 340 | 340 | 222.777 | 480 | 223.8 | 256.2 | 16.4 | 72 | 5.236 | 2.389 | 2.251 | 0.138 |
| Ramghat | Post | ORW071601111 | 09/01/2016 | 27 | 20 | 8.2 | 1226 | 910 | 10.8 | 34.6 | 9.9 | 376 | 388 | 239.76 | 490 | 244.3 | 245.7 | 33.6 | 112 | 6.232 | 2.113 | 2.034 | 0.079 |
| Ramghat | Post | ORW071601120 | 09/01/2016 | 27 | 20 | 8.2 | 1268 | 920 | 8.8 | 80.3 | 10.9 | 312 | 324 | 236.763 | 486 | 255 | 231 | 35.6 | 92 | 4.452 | 2.245 | 2.177 | 0.068 |
| Kaliyadeh | Pre | ORW071601115 | 08/01/2016 | 28 | 24 | 8.2 | 960 | 750 | 7.6 | 13.8 | 16.3 | 304 | 310 | 157.842 | 366 | 189.6 | 176.4 | 10.4 | 52 | 3.102 | 0.995 | 0.796 | 0.199 |
| Kaliyadeh | Pre | ORW071601124 | 08/01/2016 | 28 | 24 | 8.3 | 947 | 740 | 7.2 | 13.7 | 14.6 | 306 | 312 | 156.843 | 370 | 176.8 | 193.2 | 8.8 | 36 | 3.133 | 1.624 | 1.474 | 0.15 |
| Kaliyadeh | During | ORW071601116 | 09/01/2016 | 24 | 21 | 7.7 | 991 | 910 | 2.8 | 7.54 | 18.8 | 316 | 316 | 148.851 | 364 | 175 | 189 | 14 | 72 | 3.026 | 2.882 | 2.439 | 0.443 |
| Kaliyadeh | During | ORW071601125 | 09/01/2016 | 24 | 21 | 7.7 | 997 | 920 | 3.6 | 13.7 | 33.6 | 322 | 322 | 161.838 | 370 | 178.9 | 191.1 | 12.4 | 64 | 3.011 | 2.322 | 2.02 | 0.302 |
| Kaliyadeh | Post | ORW071601117 | 09/01/2016 | 27 | 21 | 7.9 | 1013 | 790 | 4.4 | 18.7 | 15.9 | 290 | 290 | 169.83 | 384 | 184.5 | 199.5 | 7.2 | 36 | 2.563 | 3.962 | 3.578 | 0.384 |
| Kaliyadeh | Post | ORW071601126 | 09/01/2016 | 27 | 21 | 7.9 | 1005 | 760 | 3.6 | 15.8 | 10.9 | 284 | 284 | 169.83 | 372 | 176.7 | 195.3 | 8 | 44 | 2.528 | 3.322 | 3.167 | 0.155 |
| Triveni Ghat | Pre | ORW081602130 | 07/02/2016 | 26 | 24 | 8.8 | 690 | 300 | 11.2 | 15.1 | 8.3 | 160 | 178 | 61.938 | 194 | 65.9 | 128.1 | 10.8 | 36 | 6.744 | 1.823 | 0.93 | 0.893 |
| Triveni Ghat | Pre | ORW081602139 | 07/02/2016 | 26 | 24 | 8.8 | 670 | 280 | 11.6 | 15.9 | 7 | 164 | 180 | 62.937 | 194 | 68 | 126 | 7.6 | 28 | 6.42 | 1.902 | 0.96 | 0.942 |
| Triveni Ghat | During | ORW081602131 | 08/02/2016 | 20 | 20 | 8.7 | 603 | 340 | 9.6 | 12.4 | 10.1 | 152 | 164 | 52.947 | 156 | 61.5 | 94.5 | 12.4 | 44 | 4.411 | 0.723 | 0.107 | 0.616 |
| Triveni Ghat | During | ORW081602140 | 08/02/2016 | 20 | 20 | 8.7 | 623 | 350 | 9.2 | 13.2 | 11.4 | 152 | 162 | 54.945 | 148 | 55.6 | 92.4 | 16.4 | 40 | 4.569 | 0.788 | 0.112 | 0.676 |
| Triveni Ghat | Post | ORW081602132 | 08/02/2016 | 28 | 22 | 8.6 | 659 | 350 | 10.4 | 13.5 | 15.6 | 158 | 170 | 48.951 | 186 | 26.4 | 159.6 | 16 | 52 | 3.378 | 0.812 | 0.118 | 0.694 |
| Triveni Ghat | Post | ORW081602141 | 08/02/2016 | 28 | 22 | 8.6 | 580 | 340 | 9.6 | 14.2 | 19.5 | 156 | 170 | 46.953 | 184 | 32.8 | 151.2 | 20 | 64 | 3.625 | 0.845 | 0.109 | 0.736 |
| Ramghat | Pre | ORW081602127 | 07/02/2016 | 25 | 23 | 8.9 | 1277 | 790 | 18 | 21.7 | 7.2 | 170 | 184 | 212.787 | 356 | 221.6 | 134.4 | 9.2 | 32 | 7.086 | 1.244 | 0.204 | 1.04 |
| Ramghat | Pre | ORW081602136 | 07/02/2016 | 25 | 23 | 8.9 | 1270 | 800 | 18.8 | 22.4 | 11.1 | 172 | 188 | 214.785 | 354 | 221.7 | 132.3 | 12.4 | 44 | 7.186 | 1.252 | 0.209 | 1.043 |
| Ramghat | During | ORW081602128 | 08/02/2016 | 24 | 21 | 8.3 | 1350 | 810 | 9.2 | 32.2 | 18.8 | 196 | 200 | 213.786 | 376 | 241.6 | 134.4 | 20.4 | 80 | 12.828 | 1.776 | 0.206 | 1.57 |
| Ramghat | During | ORW081602137 | 08/02/2016 | 24 | 21 | 8.3 | 1332 | 800 | 8.8 | 34.5 | 25.1 | 188 | 192 | 215.784 | 374 | 239.6 | 134.4 | 32.4 | 96 | 11.045 | 1.556 | 0.245 | 1.311 |
| Ramghat | Post | ORW081602129 | 08/02/2016 | 27 | 23 | 8.4 | 1442 | 810 | 10.4 | 44.5 | 22.6 | 204 | 210 | 223.776 | 366 | 256.8 | 109.2 | 30 | 108 | 12.923 | 1.987 | 0.294 | 1.693 |
| Ramghat | Post | ORW081602138 | 08/02/2016 | 27 | 23 | 8.4 | 1456 | 820 | 11.2 | 46.2 | 19.8 | 204 | 214 | 221.778 | 372 | 258.6 | 113.4 | 22 | 100 | 12.856 | 1.998 | 0.305 | 1.693 |
| Kaliyadeh | Pre | ORW081602133 | 07/02/2016 | 27 | 24 | 8.3 | 1354 | 800 | 8.8 | 9.2 | 19.3 | 332 | 340 | 56.943 | 390 | 156.9 | 233.1 | 14 | 88 | 4.756 | 1.682 | 1.579 | 0.103 |
| Kaliyadeh | Pre | ORW081602142 | 07/02/2016 | 27 | 24 | 8.3 | 1330 | 800 | 8.4 | 8.9 | 13.6 | 330 | 336 | 58.941 | 384 | 146.7 | 237.3 | 9.2 | 80 | 4.234 | 1.582 | 1.489 | 0.093 |
| Kaliyadeh | During | ORW081602134 | 08/02/2016 | 27 | 22 | 8.3 | 1372 | 750 | 8.8 | 12.7 | 39 | 356 | 364 | 153.846 | 396 | 183.9 | 212.1 | 26.4 | 84 | 3.018 | 1.025 | 0.941 | 0.084 |
| Kaliyadeh | During | ORW081602143 | 08/02/2016 | 27 | 22 | 8.3 | 1357 | 770 | 8.4 | 12.5 | 41.7 | 350 | 358 | 151.848 | 396 | 188.1 | 207.9 | 34.4 | 92 | 3.112 | 1.033 | 0.952 | 0.081 |
| Kaliyadeh | Post | ORW081602135 | 08/02/2016 | 27 | 23 | 7.7 | 1352 | 840 | 0.8 | 17.9 | 23 | 372 | 372 | 166.833 | 382 | 167.8 | 214.2 | 30 | 116 | 5.871 | 2.01 | 1.918 | 0.092 |
| Kaliyadeh | Post | ORW081602144 | 08/02/2016 | 27 | 23 | 7.7 | 1344 | 840 | 1.2 | 18.2 | 27 | 370 | 370 | 167.832 | 378 | 157.5 | 220.5 | 30 | 120 | 5.652 | 2.018 | 1.822 | 0.196 |
