# Supplementary material for: Mass bathing events in River Kshipra, Central India- influence on the water quality and the antibiotic susceptibility pattern of commensal E.coli
Source: PLoS One. 2020 Mar 4;15(3):e0229664. doi: 10.1371/journal.pone.0229664 (PMC7055887; doi:10.1371/journal.pone.0229664)
Supplement: Attachment — Submitted filename: response to reviewer.docx [file pone.0229664.s002.docx]

PONE-D-19-25307
Mass-bathing events in River Kshipra, Central India- influence on the water quality and the antibiotic susceptibility pattern of commensal *E.coli*

We are thankful to the Editor and reviewer for the comments and suggestions required for the improvement of the manuscript. Following are the reply to the various queries and concerns.

**Editors Comments**

1. When submitting your revision, we need you to address these additional requirements.
Please ensure that your manuscript meets PLOS ONE's style requirements, including those for file naming. The PLOS ONE style templates can be found at
http://www.journals.plos.org/plosone/s/file?id=wjVg/PLOSOne_formatting_sample_main_body.pdf and http://www.journals.plos.org/plosone/s/file?id=ba62/PLOSOne_formatting_sample_title_authors_affiliations.pdf

Author's Reply: Formatting done as suggested.

2. Funding Statement: The funders had no role in study design, data collection and analysis, decision to publish, or preparation of the manuscript.

Author's Reply: Added the statement.

3.  We note that you have indicated that data from this study are available upon request. PLOS only allows data to be available upon request if there are legal or ethical restrictions on sharing data publicly. For information on unacceptable data access restrictions, please see http://journals.plos.org/plosone/s/data-availability#loc-unacceptable-data-access-restrictions.
In your revised cover letter, please address the following prompts:
a) If there are ethical or legal restrictions on sharing a de-identified data set, please explain them in detail (e.g., data contain potentially identifying or sensitive patient information) and who has imposed them (e.g., an ethics committee). Please also provide contact information for a data access committee, ethics committee, or other institutional body to which data requests may be sent.
b) If there are no restrictions, please upload the minimal anonymized data set necessary to replicate your study findings as either Supporting Information files or to a stable, public repository and provide us with the relevant URLs, DOIs, or accession numbers. Please see http://www.bmj.com/content/340/bmj.c181.long for guidelines on how to de-identify and prepare clinical data for publication. For a list of acceptable repositories, please see http://journals.plos.org/plosone/s/data-availability#loc-recommended-repositories.
We will update your Data Availability statement on your behalf to reflect the information you provide.

Author's Reply: Supporting files are added.

4. We note that Figure 1 in your submission contain [map/satellite] images which may be copyrighted. All PLOS content is published under the Creative Commons Attribution License (CC BY 4.0), which means that the manuscript, images, and Supporting Information files will be freely available online, and any third party is permitted to access, download, copy, distribute, and use these materials in any way, even commercially, with proper attribution. For these reasons, we cannot publish previously copyrighted maps or satellite images created using proprietary data, such as Google software (Google Maps, Street View, and Earth).

Author's Reply: We have now replaced the previous figures and upload the map that is freely available in GADM database  ( <https://gadm.org/>) and display only three sites of sampling. The figure 1 legend now shows all details.

5. Expert reviewers have pointed out the need to revise the manuscript updating information on antimicrobial susceptibility testing methodology and controls. Do you have data on colistin susceptibility using MIC method in agar or broth dilution?

Author's Reply: As mentioned in reply to reviewer in subsequent paragraph

**Reviewer's Comments**

1. Testing the antimicrobial susceptibility for Colistin requires MIC with agar or broth microdilution, and can no longer be reported using disk diffusion.

Author's Reply: Yes, we know that antimicrobial susceptibility for Colistin requires MIC with agar or broth micro-dilution. As pointed out in the next comment, we however did the analyses some time back and therefore we have included the explanation for the same in limitation as "The study has some limitations such as colistin resistance should have been performed and confirmed by MIC method and not by disk diffusion method. We, however, simultaneously performed mcr-1 gene detection by PCR", which to extent detected in colistin resistance.

We missed to include the mcr-1gene detection in methodology in molecular method section, which we have added now.

2. Kindly justify why this study carried out in 2014-16 is submitted for publication in 2019.

Author's Reply:  As the study is a part of bigger study conducted from 2014-17, which ran over to year 2018. Thus, other things came in between. All the laboratory work, however, was done simultaneously as data were collected and thus long ago before the new requirements for colistin susceptibility testing.
